# Identification of a tumor immune-inflammation signature predicting prognosis and immune status in breast cancer

**DOI:** 10.3389/fonc.2022.960579

**Published:** 2023-01-12

**Authors:** Yajing Liu, Wenhao Ouyang, Hong Huang, Yujie Tan, Zebang Zhang, Yunfang Yu, Herui Yao

**Affiliations:** ^1^ Guangdong Provincial Key Laboratory of Malignant Tumor Epigenetics and Gene Regulation, Department of Medical Oncology, Breast Tumor Center, Phase I Clinical Trial Center, Sun Yat-sen Memorial Hospital, Sun Yat-sen University, Guangzhou, China; ^2^ School of Medicine, Guilin Medical College, Guilin, China; ^3^ Faculty of Medicine, Macau University of Science and Technology, Taipa, Macao SAR, China

**Keywords:** breast cancer, tumor immune-inflammation signature, prognostic prediction, IL27, tumor immune microenvironment

## Abstract

**Background:**

Breast cancer has become the malignancy with the highest mortality rate in female patients worldwide. The limited efficacy of immunotherapy as a breast cancer treatment has fueled the development of research on the tumor immune microenvironment.

**Methods:**

In this study, data on breast cancer patients were collected from The Cancer Genome Atlas Breast Invasive Carcinoma (TCGA-BRCA) and Molecular Taxonomy of Breast Cancer International Consortium (METABRIC) cohorts. Differential gene expression analysis, univariate Cox regression analysis, and least absolute shrinkage and selection operator (LASSO) Cox regression analysis were performed to select overall survival (OS)-related, tumor tissue highly expressed, and immune- and inflammation-related genes. A tumor immune-inflammation signature (TIIS) consisting of 18 genes was finally screened out in the LASSO Cox regression model. Model performance was assessed by time-dependent receiver operating characteristic (ROC) curves. In addition, the CIBERSORT algorithm and abundant expression of immune checkpoints were utilized to clarify the correlation between the risk signature and immune landscape in breast cancer. Furthermore, the association of IL27 with the immune signature was analyzed in pan-cancer and the effect of IL27 on the migration of breast cancer cells was investigated since the regression coefficient of IL27 was the highest.

**Results:**

A TIIS based on 18 genes was constructed *via* LASSO Cox regression analysis. In the TCGA-BRCA training cohort, 10-year AUC reached 0.89, and prediction performance of this signature was also validated in the METABRIC set. The high-risk group was significantly correlated with less infiltration of tumor-killing immune cells and the lower expression level of the immune checkpoint. Furthermore, we recommended some small-molecule drugs as novel targeted drugs for new breast cancer types. Finally, the relationship between IL27, a significant prognostic immune and inflammation cytokine, and immune status was analyzed in pan-cancer. Expression of IL27 was significantly correlated with immune regulatory gene expression and immune cell infiltration in pan-cancer. Furthermore, IL27 treatment improved breast cancer cell migration.

**Conclusion:**

The TIIS represents a promising prognostic tool for estimating OS in patients with breast cancer and is correlated with immune status.

## Introduction

Breast cancer has become the malignancy with the highest mortality rate in female patients worldwide (1). According to SEER database data from 2012 to 2018, the 5-year relative survival rate of women with localized breast cancer reached up to 99%, but this fell to 30% for patients with distant metastases. Worse, the 5-year relative survival rate of patients with metastatic triple-negative breast cancer (TNBC) dropped to 12%. The American Joint Committee on Cancer (AJCC) tumor, node, metastasis (TNM) stages and molecular subtypes are widely identified as prognostic factors in breast cancer (2). However, intra-tumoral heterogenicity and extra-tumoral microenvironment complexity frequently lead to different outcomes among patients with different breast cancer subtypes (3, 4). Therefore, a reliable predictive model is worthy of investigation to aid in the early accurate diagnosis of breast cancer and individualized treatment for breast cancer patients.

Beyond traditional chemotherapy, endocrine therapy, and targeted therapy, immune checkpoint blockade (ICB) has revolutionized the treatment of breast cancer, especially unresectable or metastatic TNBC with PD-L1 expression (5, 6). Subsequent research has demonstrated that features of the tumor immune microenvironment, including the profiling of tumor-infiltrating immune cells, the landscape of cytokines, and the expression of stimulatory and inhibitory checkpoints, significantly influence ICB response (7). Furthermore, inflammation promotion and immune escape, hallmarks of the malignant progression of cancer (8), have been found to be of great prognostic value for multiple types of cancer (9, 10). Accordingly, the inflammatory response and immune-function-related gene signature model may have a reliable predictive effect in immunotherapy and prognosis owing to its strong relationship with the immune microenvironment.

Previous studies have investigated the prognostic value of immunity markers in breast cancer. The development of bioinformatics has led to an increase in prognostic scoring models for breast cancer. A subtype termed “immunity-enhanced” was identified with high expression of the 17 immunity genes and representing an intermediate outcome between LumA and other PAM50 subtypes (11). In another study, the immune-related prognostic scores of breast cancer (IPSBC) calculated from 13 immune-related genes stratified breast cancer patients into different risk groups (AUC values fluctuating between 0.76 and 0.86 in the training set). The higher-risk group associated with worse overall outcomes were characterized with less abundance of tumor-infiltrating immune cells (12). As a mechanism of innate immunity, the inflammation signature was highly associated with the tumor immune microenvironment with prognostic value. Tumor inflammation signature (TIS) was also constructed based on an 18-gene signature measuring a pre-existing but suppressed adaptive immune response within tumors. Danahwe et al. observed that the subset of breast patients with the highest 10% of the TIS score range shows substantially improved prognosis (9). In hepatocellular carcinoma (HCC), an inflammatory response-related gene signature was validated to distinguish HCC patients with different prognoses and immune statuses. Predictive accuracy was assessed with the receiver operating characteristic (ROC) curve, and the area under the curve (AUC) reached up to 0.68 (13). However, attempt should be made to combine multiple gene signatures to optimize the prognostic model, which can improve the performance of prognostic prediction.

In this study, we aimed to construct a tumor immune-inflammation signature (TIIS) with survival prediction and immune profile differentiation based on 18 immune- or inflammation-related genes. In The Cancer Genome Atlas Breast Invasive Carcinoma (TCGA-BRCA) set, the 10-year AUC of TIIS reached 0.89, and the predictive value was also validated in the Molecular Taxonomy of Breast Cancer International Consortium (METABRIC) set. Then, we further performed functional enrichment analysis and immunome analysis in different risk groups to explore enriched pathways and immune characteristics associated with TIIS. Our prognostic model identified IL27 as a key gene, and then further analysis on the relationship between IL27 and immune signature was performed in pan-cancer, as IL27 was a significant prognostic cytokine. The migration-promoting effect of IL27 on breast cancer cells was verified in *in vitro* experiments. In general, TIIS was demonstrated to be an accurate prognostic prediction and immune classification model.

## Materials and methods

### Data acquisition and preparation

RNA-sequence profiles and the corresponding clinical information of patients with breast cancer from the TCGA-BRCA dataset and METABRIC dataset were collected through the UCSC Xena platform (https://xenabrowser.net/datapages/) (*n* = 1,069) and cBioPortal (http://www.cbioportal.org/study/summary?id=brca_metabric) (*n* = 1,903), respectively. The R package “sva” was used for batch effects of these two datasets. Clinical characteristics of the TCGA-BRCA training cohort and METABRIC validation cohort are presented in **Supplementary Table 1**.

### Acquisition of immune- or inflammation-related gene sets

Immune- or inflammation-related gene sets were extracted from the Molecular Signatures Database (MSigDB, http://www.gsea-msigdb.org/gsea/index.jsp). In general, 1,094 immune-related genes and 180 inflammation-related genes shown in **Supplementary Table 2** were selected for differential expression analysis between tumor and normal tissues.

### Differential gene expression analysis

Differential gene expression analysis for breast cancer versus normal breast tissue comparison was carried out using the two-tailed Wilcoxon rank-sum test. Genes with absolute log2 fold change (LogFC) values ≥ 1.0 and a false discovery rate (FDR)-adjusted *p*-value < 0.05 were identified as differentially expressed genes (DEGs) and listed in **Supplementary Table 3**. A total of 431 immune-related DEGs and 54 inflammation-related DEGs were selected for further Gene Ontology (GO) and Kyoto Encyclopedia of Genes and Genomes (KEGG) pathway enrichment analysis.

### Construction and validation of tumor immune-inflammation signature

After removing 34 duplicate genes and 68 genes not included in the METABRIC dataset, 383 genes were finally collected for signature construction. Univariate Cox regression analysis was performed with the “survival” package to screen the DEGs significantly associated with overall survival (OS) in the training cohort, and 49 genes met the threshold of *p*-value < 0.05. Next, the “glmnet” package was applied for the least absolute shrinkage and selection operator (LASSO) regression analysis to optimize the OS-related TIIS. Then, the prognostic TIIS was established based on 18 genes’ coefficients weighted by the multiple regression. The prognostic score (PS) of each sample can be calculated as follows: PS = sum of coefficients × expression level of DEGs. Samples in both the training and validation cohort could be separated into a high-risk or low-risk group. DEGs between the two groups were identified through Wilcoxon rank-sum test. GSEA was performed to investigate the enriched signaling pathways for these DEGs. ROC curves for 1-, 3-, 5-, and 10-year OS were drawn, and the AUC was calculated with the “survivalROC” package to measure the predictive accuracy of TIIS. The Kaplan–Meier (KM) estimate was used to make survival comparison among different risk groups with a log rank *p*-value <0.05

#### Construction of the protein–protein interaction network

The protein–protein interaction (PPI) network was constructed based on the protein interaction information retrieved from Search Tool for Retrieval of Interacting Genes (STRING, https://www.string-db.org/) with interaction score > 0.4 and FDR < 0.05.

#### Subgroup prognostic analysis

KM survival analysis was performed in the TCGA-BRCA training cohort to estimate the prognostic value of TIIS in several subgroups. Age, T stage, N stage, M stage, clinical stage, and molecular subtypes were chosen as the basis for grouping.

### Analysis of immune infiltration

The proportion of tumor microenvironment components of each sample was assessed based on the Estimation of Stromal and Immune cells in Malignant Tumor tissues using Expression data (ESTIMATE) algorithm. The generated immune score, stromal score, and ESTIMATE score reflected the infiltration levels of immune cells, stromal cells, and both, respectively. The profiling difference of 22 kinds of infiltrating immune cells between different risk groups was estimated using the CIBERSORT algorithm (https://cibersort.stanford.edu) and quantified with the Wilcoxon rank-sum test. The expression of 37 immune checkpoint molecules was also compared with the Wilcoxon rank-sum test.

### Analysis of drug sensitivity

Correlation between the expression of 18 genes of the prognostic model and drug sensitivity was evaluated through NCI-60 Analysis Tools in The CellMiner database (https://discover.nci.nih.gov/cellminer/) (14). Pearson’s correlation analysis was visualized with the “ggplot2” package.

### Analysis of association between IL27 expression and immune characteristics

Comprehensive information of pan-cancers in the TCGA dataset was obtained from the UCSC Xena platform (https://xenabrowser.net/datapages/). The expression of IL27 (cancer versus normal tissue) in pan-cancers was counted using the two-tailed Wilcoxon rank-sum test. The ESTIMATE algorithm was used to calculate the immune score, stromal score, and ESTIMATE score of each sample, and the “psych (version 2.1.6)” package was used to calculate Pearson’s correlation coefficient and assess the correlation between IL27 expression and score and estimate the relationship between IL27 expression and immune regulatory or checkpoint gene expression. Statistical graphs were drawn *via* http://sangerbox.com/.

#### Mapping tissue protein expression

The immunohistochemistry (IHC) protein expression of IL27 in human breast and liver tumor tissues and normal tissues was downloaded from the Human Protein Atlas database (https://www.proteinatlas.org/).

#### Cell culture and migration assay

MDA-MB-231 cells were obtained from the American Type Culture Collection (ATCC) and cultured according to standard protocols at 37°C under 5% CO_2_. Migration capacity was evaluated using scratch test and transwell migration assay. MDA-MB-231 cells were seeded into 12-well plates. When the cells grew to nearly 100% confluence, the monolayer cells were scratched with the tip of a 200-µl pipette and photographed. After 24 h of treatment with or without IL27 (50 ng/ml), cells were washed and photographed again. For transwell migration assay, 5×10^4^ cells suspended in 200 µl of serum-free culture medium were seeded into the upper chamber (Corning Incorporated, NYC, USA), and the lower compartment was filled with 600 µl of culture medium containing 10% fetal bovine serum with or without IL27 (50 ng/ml). After incubation for 24 h, the incubation medium and the cells on the upper surface of membrane were wiped off. Cells on the underside of the membrane were fixed in 4% paraformaldehyde, stained with 0.1% crystal violet, and photographed under an inverted microscope at 100× magnification.

### Statistical analysis

The Wilcoxon rank-sum test was employed to explore differences between two groups. R software (www.r-project.org, version) was used for all data analysis and visualization. A *p*-value < 0.05 was considered statistically significant.

## Results

### Clinical characteristics

**Figure 1:**
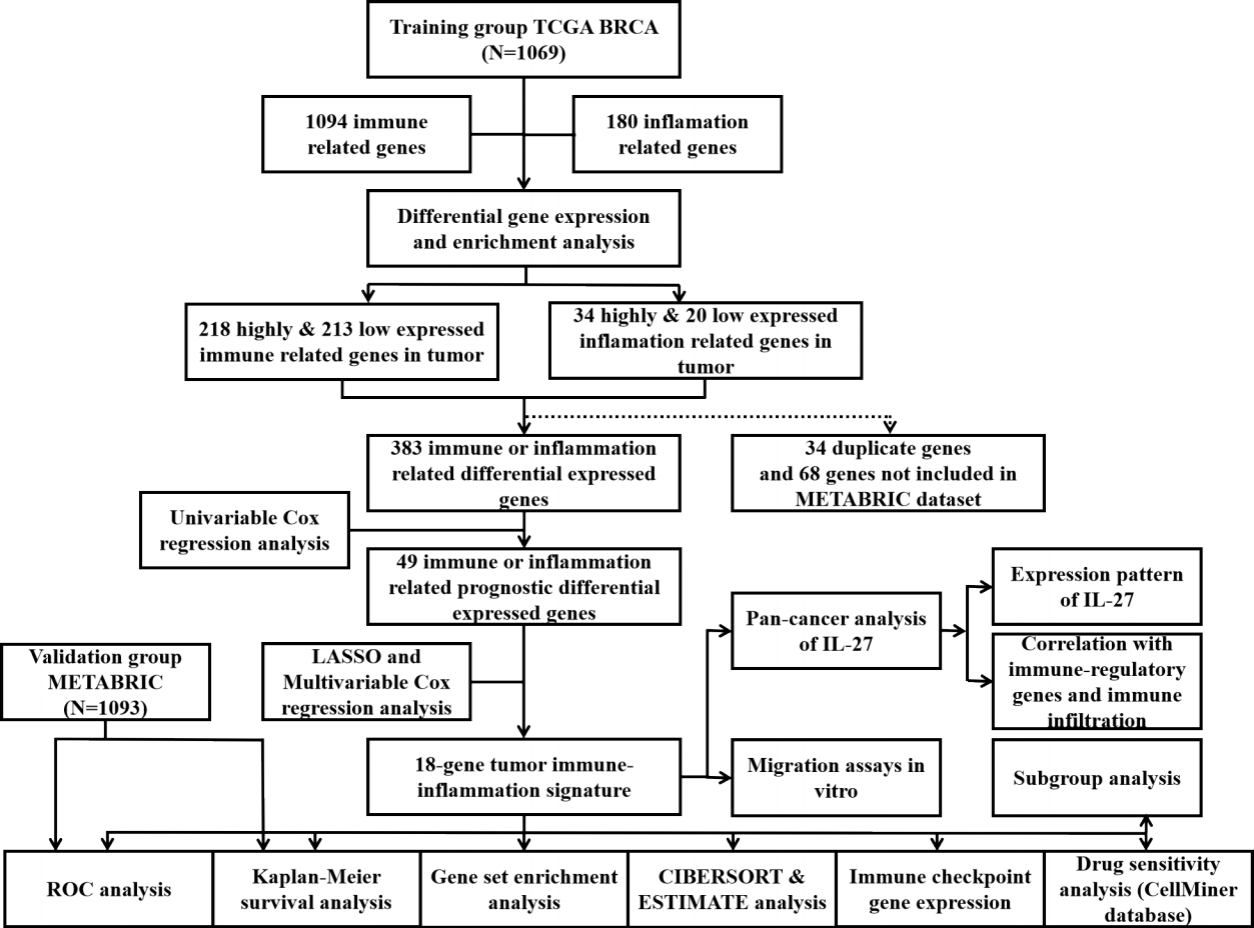
Flowchart of this study.

As shown in the flowchart of this study in **Figure 1**, a total of 1,069 and 1,903 breast cancer patients were recruited from the TCGA-BRCA and METABRIC cohorts, respectively, along with detailed follow-up data, clinical stages, and personal information. As shown in **Supplementary Table 1**, the mean OS was 41.87 months in the TCGA-BRCA set and 125.19 months in the METABRIC set. Most cases in TCGA-BRCA were in clinical stage II or II.

### Identification of immune- or inflammation-related differentially expressed genes

**Figure 2:**
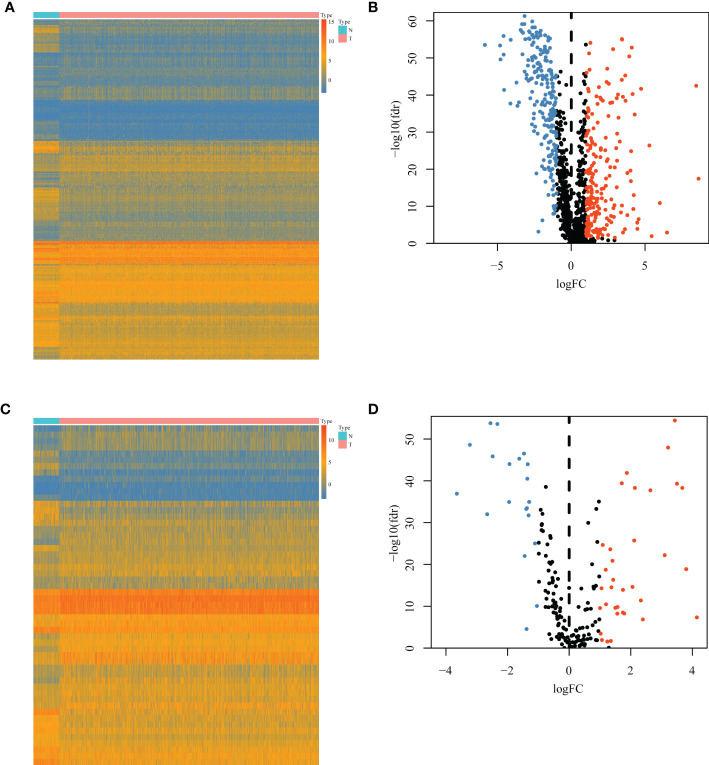
Differential immune or inflammation-related gene expression analysis in the TCGA-BRCA training cohort. **(A)** Heatmap and **(B)** volcano plot of 1,094 immune-related genes in the TCGA-BRCA training cohort showing differential expression between cancer and normal samples. **(C)** Heatmap and **(D)** volcano plot of 180 inflammation-related genes in the TCGA-BRCA training cohort identifying DEGs between cancer and normal tissues. N, normal tissues; T, tumor tissues; DEGs, differentially expressed genes.

**Figure 3:**
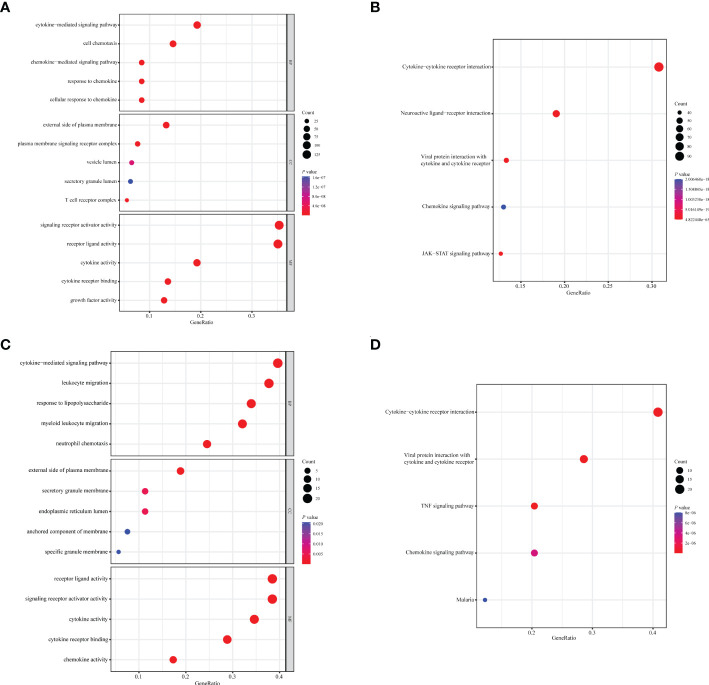
Enrichment analysis of immune- or inflammation-related differentially expressed genes in the TCGA-BRCA training cohort. Bubble plot of **(A)** GO and **(B)** KEGG pathway analysis of immune-related DEGs. Bubble plot of **(C)** GO and **(D)** KEGG pathway analysis of inflammation-related DEGs. Each graph showed the top five enriched pathways of BP, CC, MF, and KEGG. GO, Gene Ontology; KEGG, Kyoto Encyclopedia of Genes and Genomes; DEGs, differentially expressed genes; BP, biological process; CC, cellular components; MF, molecular function. Gene ratio: the ratio of enriched genes to the total number of genes in the relative pathway in the corresponding database. Count: the number of enriched DEGs in each pathway.

A total of 1,094 immune-related genes and 180 inflammation-related genes were extracted from MSigDB (**Supplementary Table 2**). Among them 431 immune-related genes (**Figures 2A, B**) and 54 inflammation-related genes (**Figures 2C, D**) reached the criteria of LogFC ≥ 1.0 and *p*-value < 0.05 by the two-tailed Wilcoxon rank-sum test. Immune-related DEGs were linked to pathways involved in the cytokine-mediated signal pathway, cell chemotaxis, and cytokine–cytokine interaction (**Figures 3A, B**). Similarly, inflammation-related DEGs were enriched in pathways including cytokine-mediated signal pathway, response to lipopolysaccharide, and immune cell migration (**Figures 3C, D**).

### Identification of tumor immune-inflammation signature

**Figure 4:**
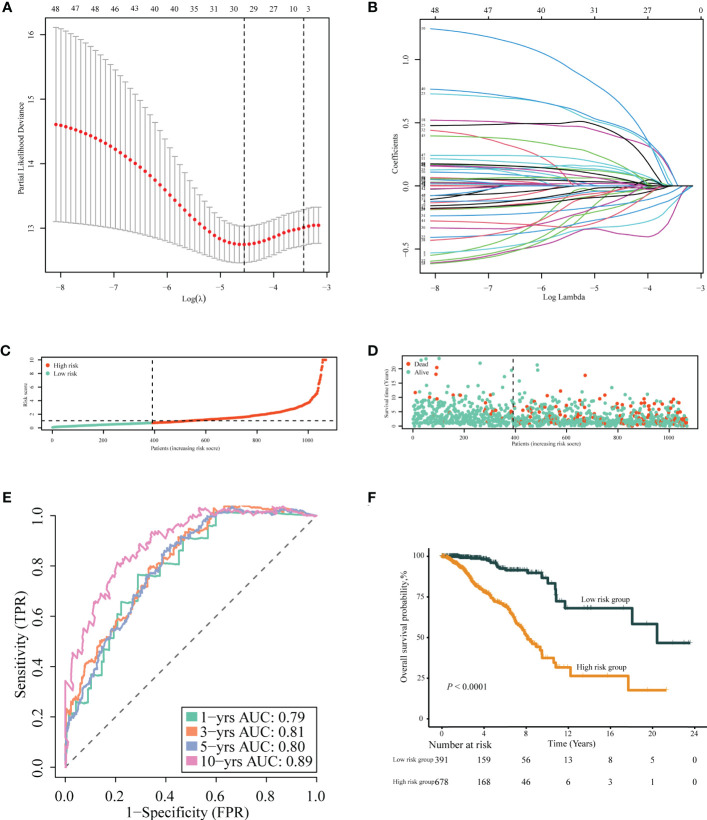
Construction of TIIS. **(A, B)** LASSO Cox regression of differentially expressed immune- or inflammation-related genes in the TCGA-BRCA training cohort. The risk curve of the distribution of patients’ prognostic score **(C)**, survival time, and status **(D)**. Time-dependent ROC curves **(E)** for survival probability of TIIS. Kaplan–Meier curves **(F)** for 1,069 patients classified as high and low risk by TIIS. LASSO, least absolute shrinkage and selection operator; ROC, receiver operating characteristic. TIIS, tumor immune-inflammation signature.

After removing 34 duplicate genes and 68 genes not included in the METABRIC dataset, 383 genes were finally selected for analysis (**Supplementary Figure 1**), including 183 upregulated genes in tumor tissues and 200 upregulated genes in normal tissues. Univariable Cox regression analysis was performed to screen immune- or inflammation-related DEGs associated with OS. Then, 49 genes were identified as survival-associated DEGs, which are listed in **Supplementary Table 4**, for further screening *via* LASSO regression analysis. Ultimately, 18 DEGs were selected for prognostic model construction (**Figures 4A, B**), with corresponding regression coefficients determined by multivariable Cox regression analysis, as shown in **Supplementary Table 5**. The obtained TIIS-based prognostic score was calculated as follows: (PS) = ∑coefficient ×gene expression = PSME2 × (−0.43458) + IL27 × 1.034941 + NRP3 × 0.166836 + TSLP × (−0.57442) + NOS1 × 0.496469 + APOD × (−0.1144) + ADRB1 × (−0.36949) + LCN1 × 0.565002 + HGF × 0.761114 + JUN × (−0.19551) + IFNG × (−0.46269) + FABP6 × 0.161865 + FLT3 × (−0.19532) + KCNMB2 × 0.716553 + NRG1 × (−0.44158) + AVPR1A × 0.462009 + AMH × 0.288371 + SDC1 × 0.147517. According to this immune-inflammation signature formula, patients with a higher risk score were classified into the high-risk group (*n* = 678) with more deaths, and the remaining patients with a lower risk score made up the low-risk group (*n* = 391) with more surviving cases (**Figures 4C, D**). A PPI network of the selected 18 genes was constructed based on the STRING database, indicating the broad functional overlap between IL27 and IFN-γ, and interaction in remodeling the tumor immune microenvironment between IFN-γ and HGF (**Supplementary Figure 2**). In addition, the hazard ratio of six genes as independent protective factors and six genes as independent risk factors of prognosis is listed in the forest plot (**Supplementary Figure 3**).

### Evaluation of TIIS with predictive efficacy

**Figure 5:**
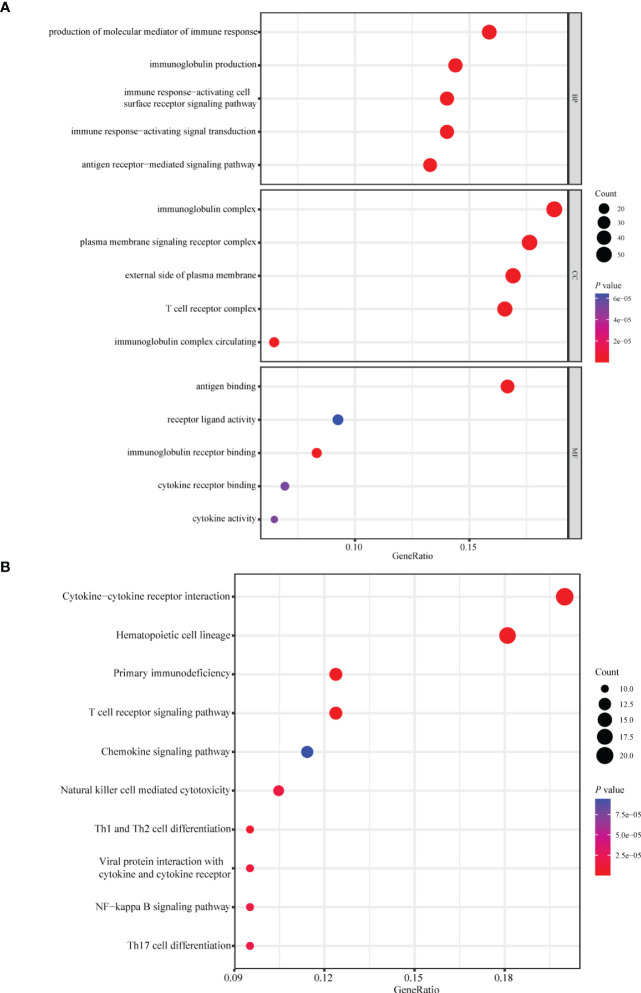
Enrichment analysis of differentially expressed genes between the high- and low-risk group. Bubble plot of **(A)** GO and **(B)** KEGG pathway analysis of genes expressed differentially between the high- and low-risk group. GO, Gene Ontology; KEGG, Kyoto Encyclopedia of Genes and Genomes; BP, biological process; CC, cellular components; MF, molecular function.

Patients with breast cancer from the TCGA-BRCA training cohort or the METABRIC validation cohort were stratified into the high- or low-risk group according to the optimal cutoff value of PS determined by the “survminer” package. KM survival plots showed that patients in the high-risk group had poorer OS compared with those in the low-risk group in both cohorts. ROC graphs were created to compare the effect of the prognostic classification model in both training and validation cohorts. In the TCGA-BRCA training cohort, the 1-year, 3-year, 5-year, and 10-year AUCs were 0.79, 0.81, 0.80, and 0.89, respectively (**Figures 4E, F**). TIIS achieved higher accuracy than single risk gene predictive models (**Supplementary Figure 4A**). Moreover, in the METABRIC validation cohort, patients with a higher PS were assigned to the high-risk group and survived for a shorter period than those in the low-risk group (**Supplementary Figure 5B**). DEGs generated between the high- and low-risk groups were mainly enriched in cytokine–cytokine receptor interaction, immune response, and immunoglobulin complex formation (**Figures 5A, B**).

### Evaluation of TIIS in several subtypes of breast cancer

The predictive performance of TIIS was further investigated in several subtypes of breast cancer. TIIS exhibited excellent predictive capacity to classify patients into high and low risk for OS in both patients older than 65 and younger than 65 (**Supplementary Figure 6A**), with breast cancer larger or smaller than 5 cm (**Supplementary Figure 6B**), with lymph node metastasis or not (**Supplementary Figure 6C**), and with distant metastasis or not (**Supplementary Figure 6D**). Similarly, TIIS was also prognostic in patients with clinical stage I–III breast cancer (**Supplementary Figure 6E**), or luminal A, luminal B, and basal-like breast cancer (**Supplementary Figure 6F**). However, subgroups with stage IV or HER2-positive breast cancer did not present statistical evidence of association between TIIS and OS, which might result from insufficient sample size.

### Evaluation of TIIS with the immune landscape

**Figure 6:**
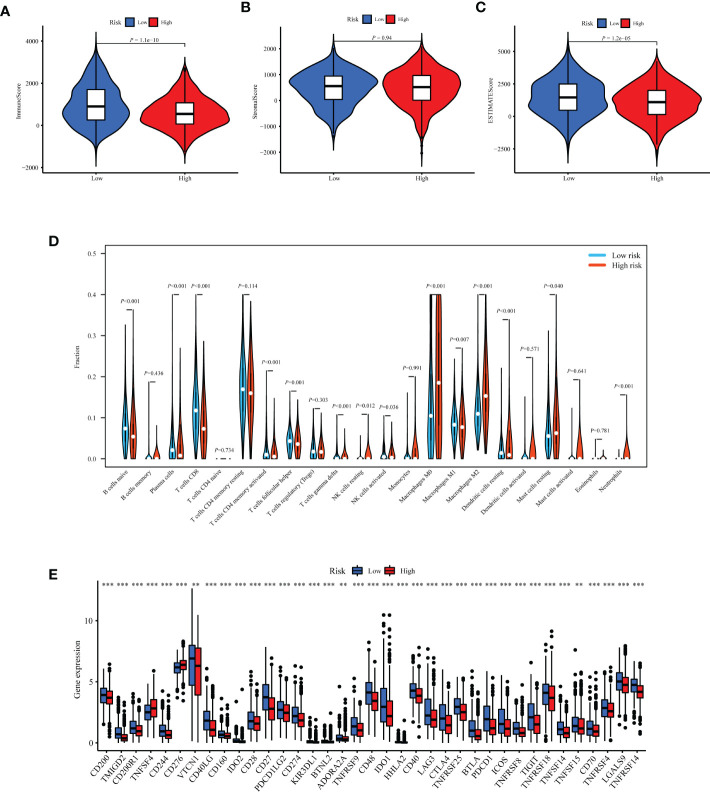
Exploration of differences in the tumor immune microenvironment between the high- and low-risk groups of the TCGA-BRCA training cohort. Immune score **(A)**, stromal score **(B)**, and tumor purity **(C)** were evaluated using ESTIMATE analysis and compared between high- and low-risk groups. **(D)** Infiltration of 22 kinds of immune cells were analyzed by the CIBERSORT algorithm. **(E)** Expression of 37 immune regulatory genes were compared between the high- and low-risk groups. **p < 0.01, ***p < 0.001.

Based on the prognostic value of TIIS, the immune landscape of the two risk groups was systematically compared. Lower immune cell infiltration and higher tumor purity were found in high-risk samples (**Figures 6A–C**). Furthermore, types of tumor-infiltrating immune cells were profiled with the CIBERSORT algorithm. The proportion of M2 macrophages was higher in the high-risk group. In addition, the infiltration of M1 macrophages, naive B cells, plasma cells, CD8-positive T cells, and resting dendritic cells was significantly greater in the low-risk group (**Figure 6D**). These results were consistent with those of previous reports on the tumor immune microenvironment, revealing that tumor-killing immune cells tended to be more common in the low-risk group and *immunosuppressive tumor microenvironment* was associated with poorer prognosis (15). Besides the distinct distributive pattern of immune cells, expression of genes involved in immune checkpoints or activation was also different. The immune checkpoint gene expression including PD-L1 (CD274), CTLA-4, TIGHT, and LAG3 decreased significantly in the high-risk group, indicating a poor immunotherapy response (**Figure 6E**). In summary, the classification method based on TIIS could distinguish between patients with different prognoses due to different tumor immune microenvironments.

### Drug sensitivity analysis of genes comprising TIIS

**Figure 7:**
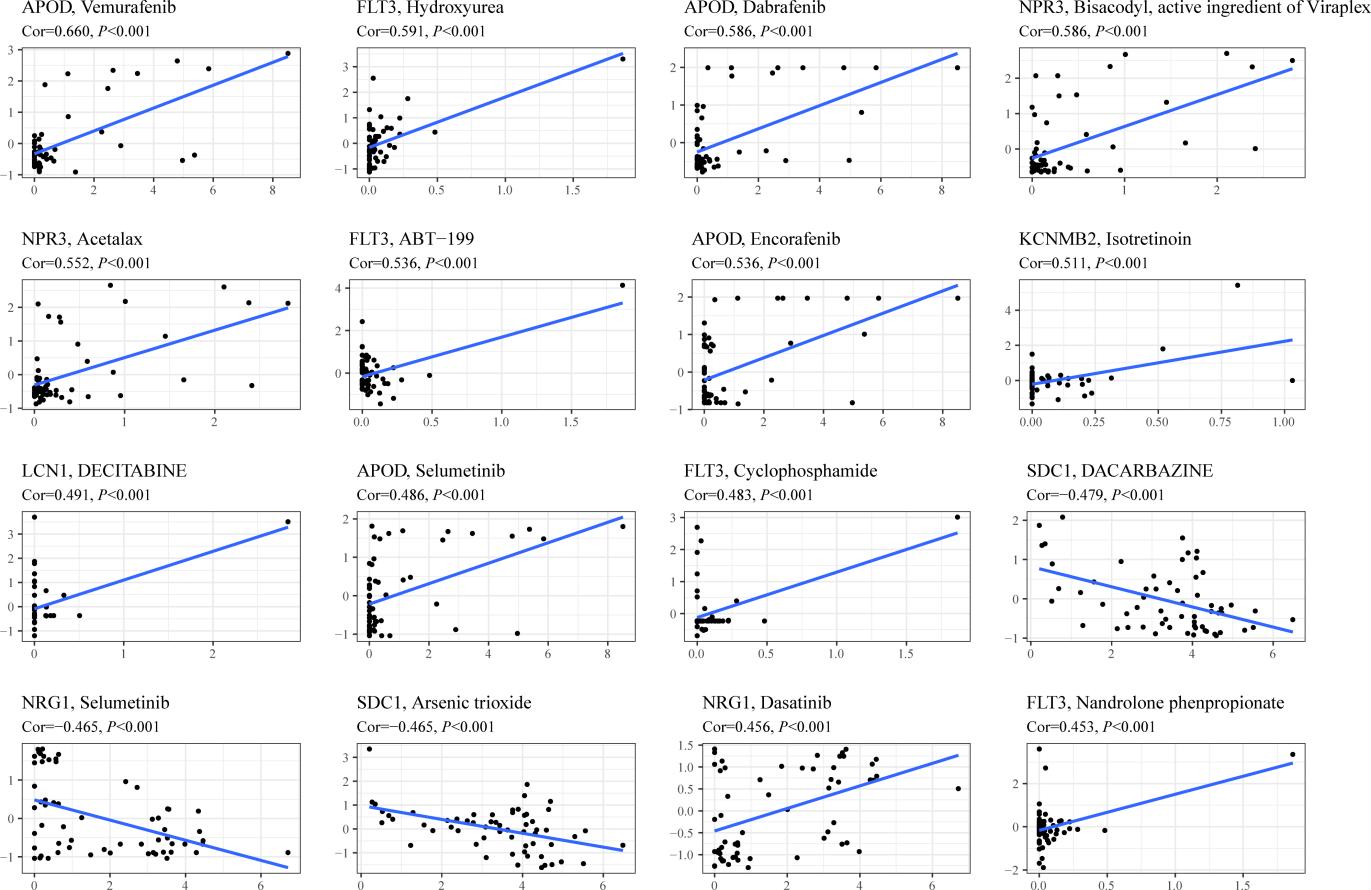
Drug sensitivity prediction based on TIIS. Scatter plots of correlation between gene expression (abscissa) and *Z* score of anti-tumor drugs calculated by Pearson correlation test (ordinate) based on the CellMiner database. Each black point represented an independent sample. The blue line was the linear regression line.

To investigate the potential association between TIIS and drug susceptibility, Pearson correlation analysis was conducted between mRNA expression of 18 prognostic DEGs in the NCI-60 cell line and drug *Z* scores achieved from the CellMiner database. High apolipoprotein D (APOD) expression, which was negatively correlated with PS, was associated with increased sensitivity of vemurafenib (inhibitor of the B-Raf enzyme), selumetinib (a selective inhibitor of MEK), dabrafenib (inhibitor of the B-Raf enzyme), and encorafenib (inhibitor of the B-Raf enzyme). In addition, the fms-related receptor tyrosine kinase 3 (FLT3), which preferred high expression in the low-risk group, also enhanced the sensitivity of hydroxyurea (inhibition of ribonucleoside diphosphate reductase), ABT-199 (BCL-2-selective inhibitor), cyclophosphamide (alkylating agents), and nandrolone phenpropionate (an androgen and anabolic steroid medication). Conversely, high Syndecan 1 (SDC1) expression was found to develop resistance to arsenic trioxide and dacarbazine, which was consistent with its positive relationship with poor prognosis (**Figure 7**). In general, TIIS displayed intricate links with varied drug sensitivities, which may explain the ideal prognostic capacity of TIIS.

### Pan-cancer analysis of association between IL27 expression and immune characteristics

**Figure 8:**
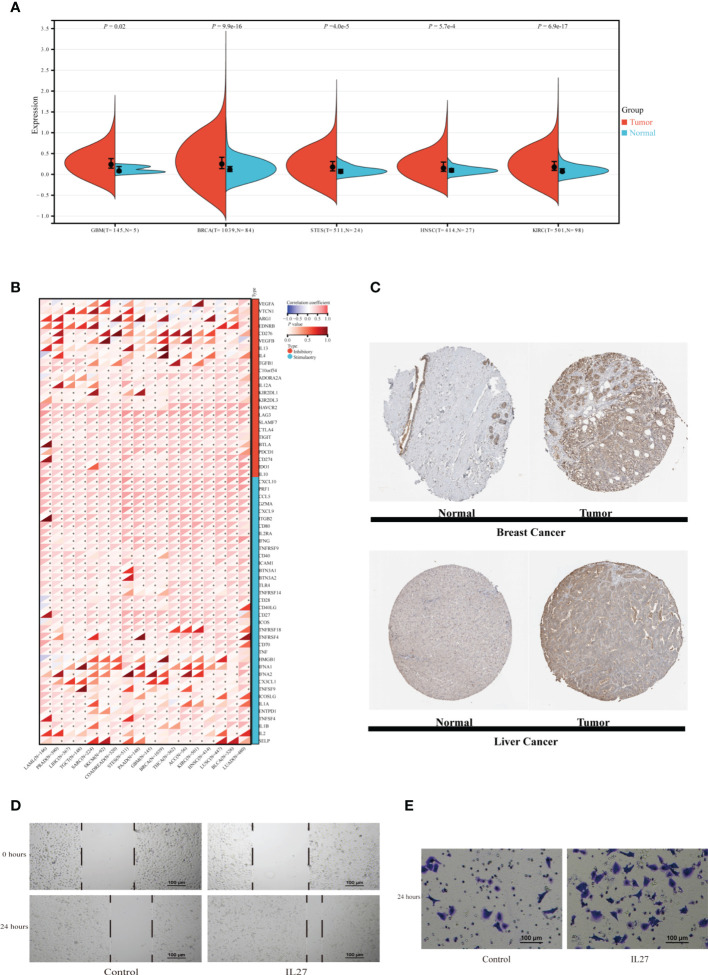
Exploration of IL27 expression characteristics and its association with the immune landscape in pan-cancer. **(A)** The expression of IL27 was compared between tumor tissue and normal tissue in different types of cancer in the TCGA database. **(B)** The correlation between expression of IL27 and immune regulatory gene expression analyzed using Pearson correlation test among 18 types of cancer. *p < 0.05. **(C)** Protein expression of IL27 in normal and tumor tissues obtained from Human Protein Atlas. MDA-MB-231 cells were incubated with or without IL27 (50 ng/ml) for 24 h. Scratch test **(D)** and transwell migration assay **(E)** of MDA-MB-231. GBM, glioblastoma multiforme; BRCA, breast invasive carcinoma; STES, stomach and esophageal carcinoma; HNSC, head and neck squamous cell carcinoma; KIRC, kidney renal clear cell carcinoma; LAML, acute myeloid leukemia; PRAD, prostate adenocarcinoma; LIHC, Liver hepatocellular carcinoma; TGCT, testicular germ cell tumors; SARC, sarcoma; SKCM, skin cutaneous melanoma; COADREAD, colon adenocarcinoma or rectum adenocarcinoma; EC, esophageal carcinoma; PAAD, pancreatic adenocarcinoma; THCA, thyroid carcinoma; ACC, adrenocortical carcinoma; LUSC, lung squamous cell carcinoma; BLCA, bladder urothelial carcinoma; LUAD, lung adenocarcinoma; T, tumor tissue; N, normal tissue.

TIIS finally consisted of 18 prognostic DEGs, and IL27 was the DEG with the highest coefficient value and hazard ratio (hazard ratio 2.81; 95% CI 1.60–4.96; *p* < 0.001) (**Supplementary Figure 3**). Breast cancer patients with higher IL27 expression had considerably worse OS than those with lower IL27 expression (**Supplementary Figure 4B**). Then, IL27 expression was compared between tumor and normal tissues in pan-cancer. As shown in **Figure 8A**, the mRNA expression of IL27 was higher in breast cancer, glioblastoma multiforme, stomach and esophageal carcinoma, kidney renal clear cell carcinoma, and head and neck squamous cell carcinoma than in corresponding normal tissues. **Figure 8C** shows the protein expression of IL27 in breast cancer and liver cancer. Consistent with RNA expression data, the IHC staining image demonstrated that IL27 protein levels were higher in breast cancer tissue compared with normal tissue. Also in tissues acquired from patients with liver cancer, protein expression of IL27 slightly increased in cancer tissue. Next, associations between IL27 and immune regulator gene expression were analyzed with Pearson’s correlation coefficient and displayed in **Figure 8B**. IL27 showed a strong positive relationship with PD-L1 (CD274), CTLA-4, TIGHT, LAG3, IFNG, TNF, and ICAM1 in various types of cancer. Furthermore, immune infiltration analysis revealed that IL27 was positively correlated with a more stromal component in lung squamous cell carcinoma, skin cutaneous melanoma, glioma, and acute myeloid leukemia (**Supplementary Figure 7A**). Additionally, IL27 expression had a higher association with immune cell infiltration and tumor purity in glioma, skin cutaneous melanoma, kidney renal carcinoma, bladder urothelial carcinoma, lung squamous cell carcinoma, acute myeloid leukemia, stomach and esophageal carcinoma, and head and neck squamous cell carcinoma (**Supplementary Figures 7B, C**).

#### Validation of the effect of IL27 on migration of breast cancer cells

IL27, as a member of the IL12 cytokine family, is secreted by antigen-presenting cells and regulates inflammatory response through JAK-STAT and p38 MAPK pathways (16, 17). In addition to reengineering tumor microenvironment, IL27 also directly affects malignant progression of cancer cells. Yan et al. also found that IL27 facilitated proliferation of breast cancer (18). As TGFB1 (19), IL10 (20), CXCL10 (21), etc., which exhibited expression association with IL27, have all been reported to affect the migration of breast cancer, we supposed that IL27 might affect the ability of breast cancer cells to migrate. In this section, migration assays were performed to examine the effect of IL27 on the migration capacity of the TNBC cell line MDA-MB-231. Scratch test showed that MDA-MB-231 treated with recombinant IL27 possessed increased migration ability (**Figure 8D**). Similarly, in the transwell migration assay, IL27 exposure improved the capacity of MDA-MB-231 to migrate through the membrane (**Figure 8E**). These results illustrated that IL27 raised breast cancer cell migration, which may partially explain the role of IL27 in poor prognosis in breast cancer.

## Discussion

Although immunotherapy has exhibited encouraging and durable efficacy in a variety of tumors, especially in patients with microsatellite instability-high (MSI-H) or mismatch repair-deficient (dMMR) solid tumors (22), ICB was just approved for advanced TNBC with positive PD-L1 (5, 6). This highlights the significance of tumor immune microenvironment analysis in predicting the prognosis and responsiveness of ICB. Shuning Ding et al. have constructed a novel immune-related prognostic signature (IRPS) with four immune-related genes (CCL1, VGF, TSLP, and FABP9). AUCs of nomogram integrating IRPS and clinical factors were 0.701 at 3 years and 0.694 at 10 years (23). Another 15-immune gene risk score model established by Chen et al. showed considerable AUC value in the training set (5-year OS AUC = 0.752) (24). Moreover, the AUC value of the nomogram constructed based on a hypoxia-immune related risk score (HIRS) and clinicopathological features to predict OS was 0.726 in the METABRIC training set (25). The AUC value of the published prediction model was hardly higher than 0.8. As inflammation plays an essential role in involving the innate immune response and shaping the ensuing adaptive immune response, prognostic models based on both immune- and inflammation-related genes may achieve superior accuracy.

Several studies have attempted to combine immune and inflammatory signatures for model construction. Studies revealed that prognosis in patients with colon cancer can be predicted by combining analysis of local infiltration of chronic inflammatory cells and systemic inflammatory responses (26). The high systemic immune-inflammation index (SII) was related to a poor prognosis in non-small cell lung cancer (NSCLC) (27) and also an independent worse prognostic factor for DFS (HR, 4.530; 95% CI, 3.279–6.258; *p* < 0.001) and OS (HR, 3.825; 95% CI, 2.594–5.640; *p* < 0.001) in breast cancer (28). ROC analysis showed that the AUC of SII for predicting DFS and OS in breast cancer was 0.724 (*p* < 0.001; 95% CI, 0.679–0.770) and 0.703 (*p* < 0.001; 95% CI, 0.645–0.761), respectively. In contrast, our study enrolled more patients in the training set (1,069 *vs*. 784), obtaining a higher AUC for OS prediction (0.89 *vs*. 0.70), and completed external validation. Similarly, our TIIS also demonstrated excellent prognostic power in different breast cancer molecular subtypes. In conclusion, we developed a more accurate immune-inflammation signature for prognostic prediction in breast cancer.

Among the 18 prognosis-related genes finally screened, IL27 was the one with the largest parameter. IL27 is usually expressed by antigen-presenting cells with pro- and anti-inflammatory effects (29). Such dual roles make investigation of effects of the IL27 in the tumor immune environment a challenge. In the pancreatic cancer preclinical model, IL27 production induced T-cell exhaustion, resulting in resistance to immunotherapy. As for HCC, IL27R signaling within the tumor microenvironment restrained the cytotoxicity of innate cytotoxic lymphocytes, while in lung cancer, IL27 treatment increased sensitivity to cisplatin in A54916. In addition, elevated IL27 expression could induce an enhanced immune response and pyroptosis (*R* = 0.64, *p* = 1.2e−55), autophagy (*R* = 0.37, *p* = 7.1e−17), and apoptosis (*R* = 0.47, *p* = 1.1e−27) in patients with melanoma (30–34). The relationship between IL27 and immune signature was analyzed in pan-cancer in this research. In breast cancer tissues, IL27 was expressed more abundantly compared with normal breast tissues. In addition, expression of IL27 was positively correlated with immune regulatory gene expression and the immune score, stromal score, and ESTIMATE score in pan-cancer. Such a strong association indicated that IL27 may be a potential predictor for ICB efficacy.

During the process of drug sensitivity analysis, the association between expression of APOD and sensitivity of B-Raf inhibitor caught our attention. Patients with high APOD expression seemed to benefit from a series of B-Raf inhibitors (e.g., vemurafenib, dabrafenib, and encorafenib, shown in **Figure 7**). APOD is an extracellular glycoprotein involved in complicated immune response (35). Recent research found that the inhibitor of B-Raf (a member of Raf family of serine/threonine protein kinases) exhibited increased tumor immune infiltration (36), and the combination of ICB and B-Raf inhibitor showed improved antitumor activity in BRAF V600E-mutant melanoma (37–39). In addition, B-Raf, as a key mediator of KRAS, was reported to drive tumor immune suppression (40). However, there is no reasonable molecular mechanism revealing the secrets between B-Raf and tumor immune characteristics or the relationship among APOD, B-Raf, and the tumor immune microenvironment. Therefore, APOD expression may be a potential biomarker for the B-Raf inhibitor combined with ICB therapy, and the mechanism of such a correlation deserves further study.

This study still has several limitations. First, TIIS did not acquire acceptable accuracy in the METABRIC validation cohort. Mildly unsatisfactory AUC in the METABRIC external validation set (10-year AUC reaching 0.55) might result from platform bias as METABRIC used microarray expression and TCGA RNA-seq (**Supplementary Figure 5A**). Different microarray protocols and geographical variations between databases could explain reasonable inter-cohort bias. Second, TIIS was established and validated with data from public databases, lacking multicenter prediction research and prospective study. Third, beyond promoting migration, the mechanism by which IL27 acted as a poor prognostic biomarker in breast cancer remained unclear. The feasibility of IL27 being a drug target for breast cancer needed to be experimentally verified.

In sum, this study integrated immune- and inflammation-related genes and defined TIIS as a new prognostic model, which was proved to predict breast cancer patients’ survival and was confirmed to be valuable in functional enrichment analysis and the tumor immune landscape. More clinical trial data may help validate the predictive value of TIIS for immunotherapy efficacy and the ability to provide new targets.

## Conclusion

In general, this study defined a prognostic model consisting of 18 immune- and inflammation-related genes. This model exhibited satisfactory predictive performance in both the TCGA-BRCA training cohort and the METABRIC validation cohort. Patients with breast cancer were successfully assigned into the high- or low-risk group, with survival times differing significantly between the two groups. Furthermore, analysis of the immune landscape between the high- or low-risk group revealed lower tumor-killing immune cell infiltration and lower immune checkpoint expression in the high-risk group. Additionally, genes that make up the signature were correlated with various drug sensitivities. Lastly, pan-cancer analysis of IL27 revealed that expression of IL27 was higher in tumor tissues and associated with a higher ESTIMATE score and expression of immune regulatory genes. In breast cancer, IL27 increased the migration ability of MDA-MB-231, which partly explained the mechanism of IL27 as a poor prognostic biomarker in breast cancer.

## Data availability statement

The original contributions presented in the study are included in the article/**Supplementary Material**. Further inquiries can be directed to the corresponding authors.

## Author contributions

Drs. YL, WO and HH are co-first authors. Concept and design: Drs. HY and YY. Data acquisition and correction: Drs. WO and HH. Data bioinformatic and statistical analysis: All authors. Migration assays performing: Drs. WO and YL. Figure visualization and manuscript writing: Drs. YL, HH and YY. Critical revision of the manuscript for important intellectual content: All authors. Obtained funding: Drs. HY and YY. Technical or material support: Drs. YY and HY. All authors contributed to the article and approved the submitted version.
