# Supplementary figures and images for: Identification of a tumor immune-inflammation signature predicting prognosis and immune status in breast cancer
Source: Front Oncol. 2023 Jan 12;12:960579. doi: 10.3389/fonc.2022.960579 (PMC9881411; doi:10.3389/fonc.2022.960579)

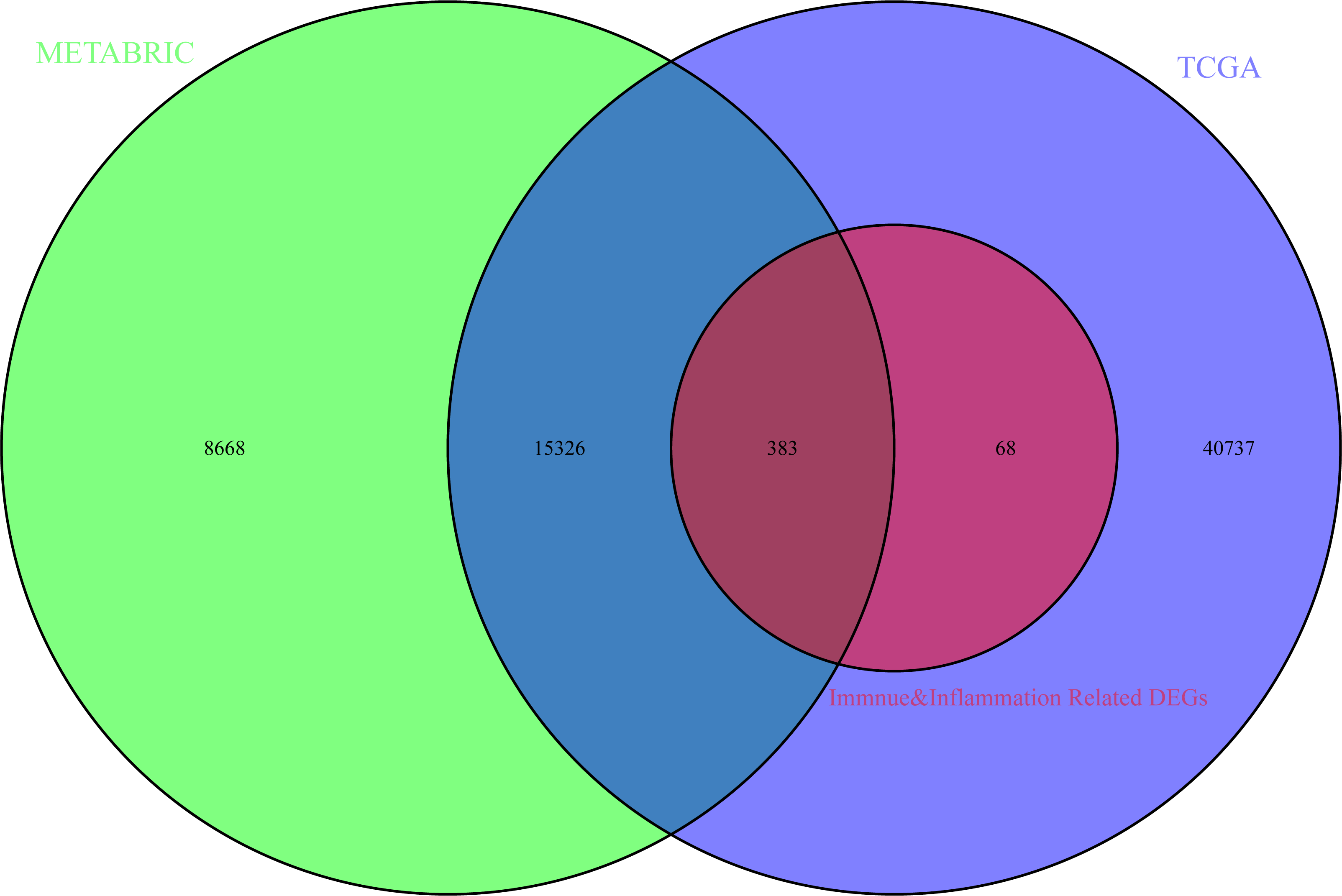

Supplement: Supplementary file 1 [file Image_1.tif]

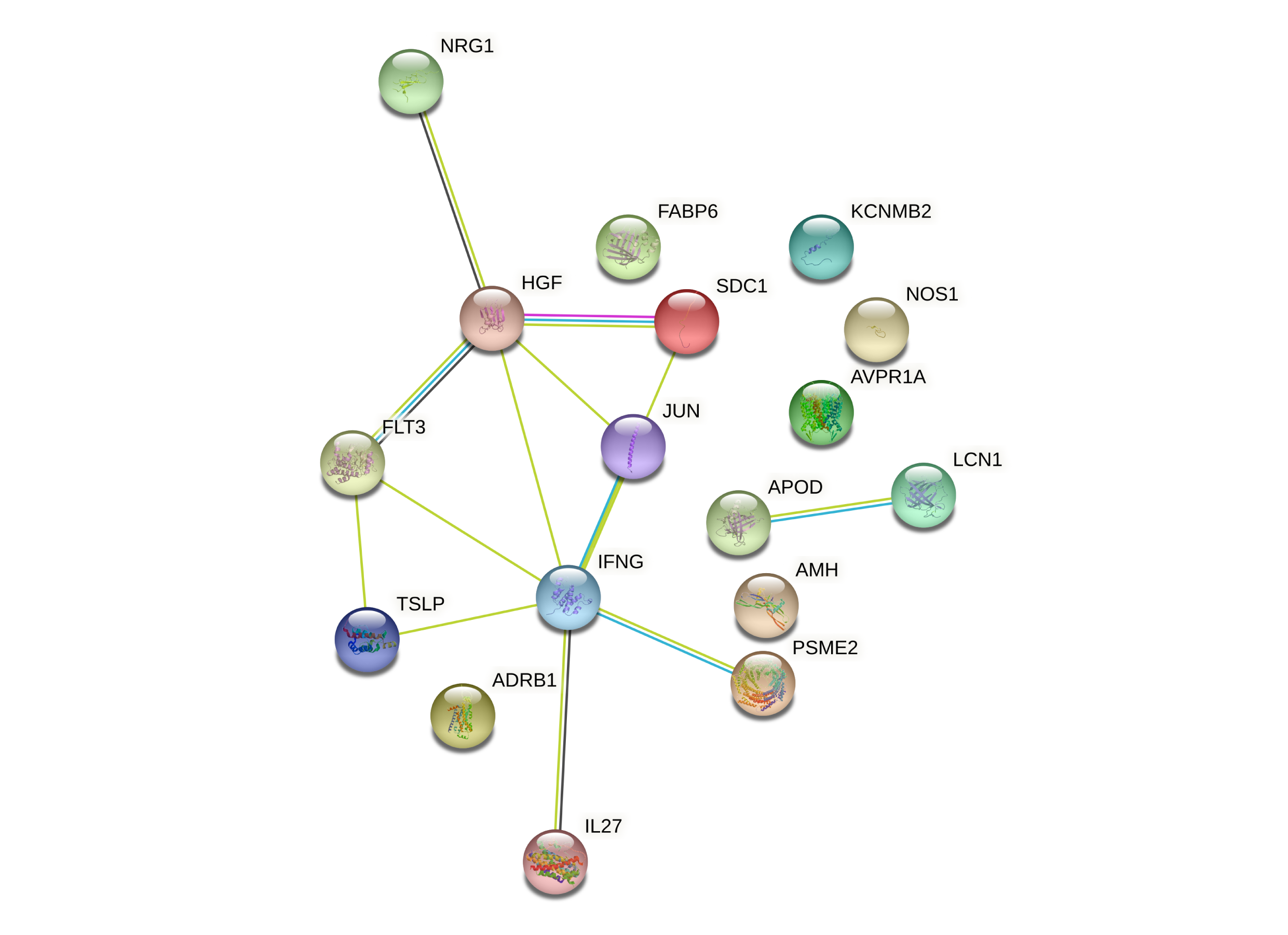

Supplement: Supplementary file 2 [file Image_2.tif]

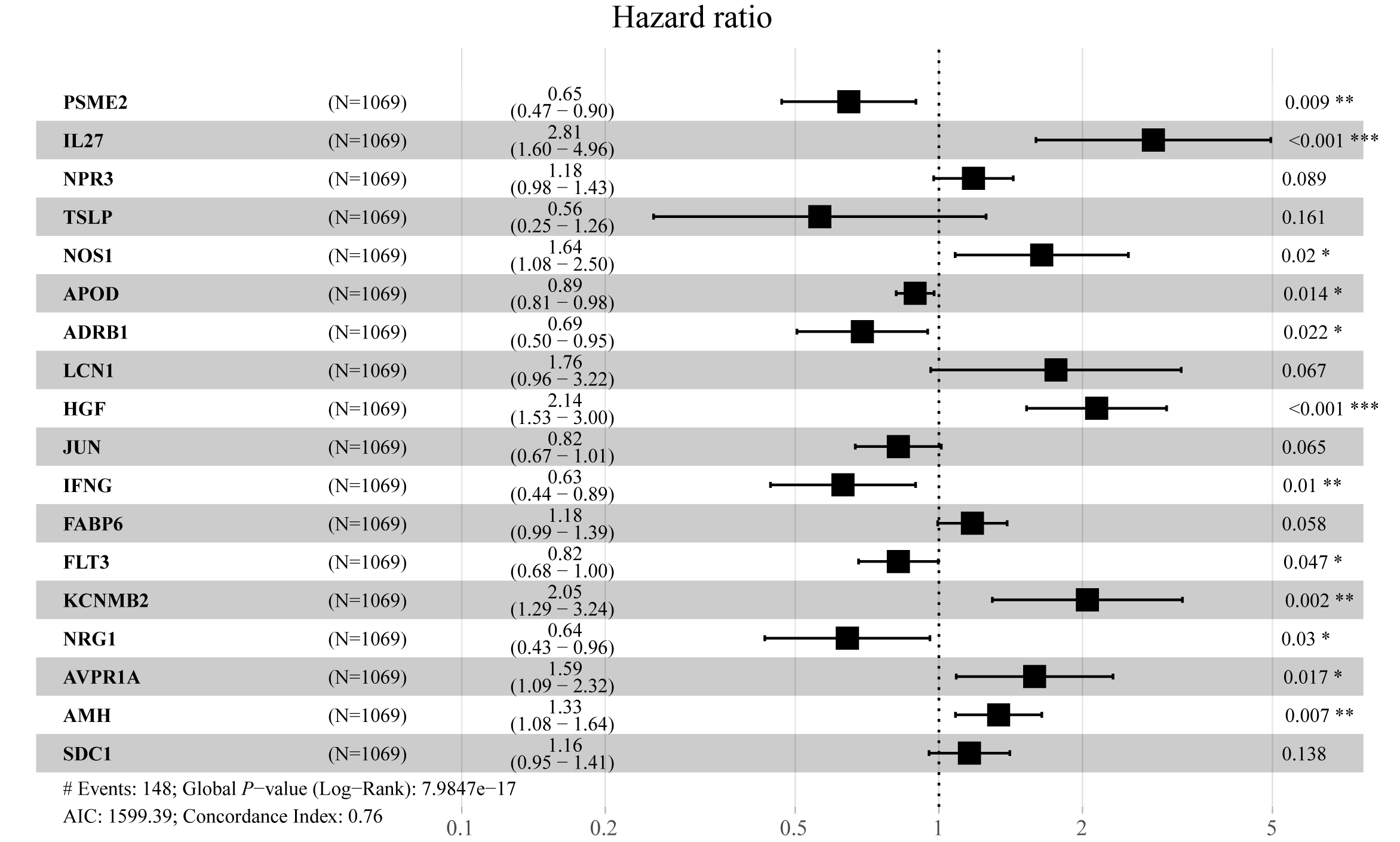

Supplement: Supplementary file 3 [file Image_3.tif]

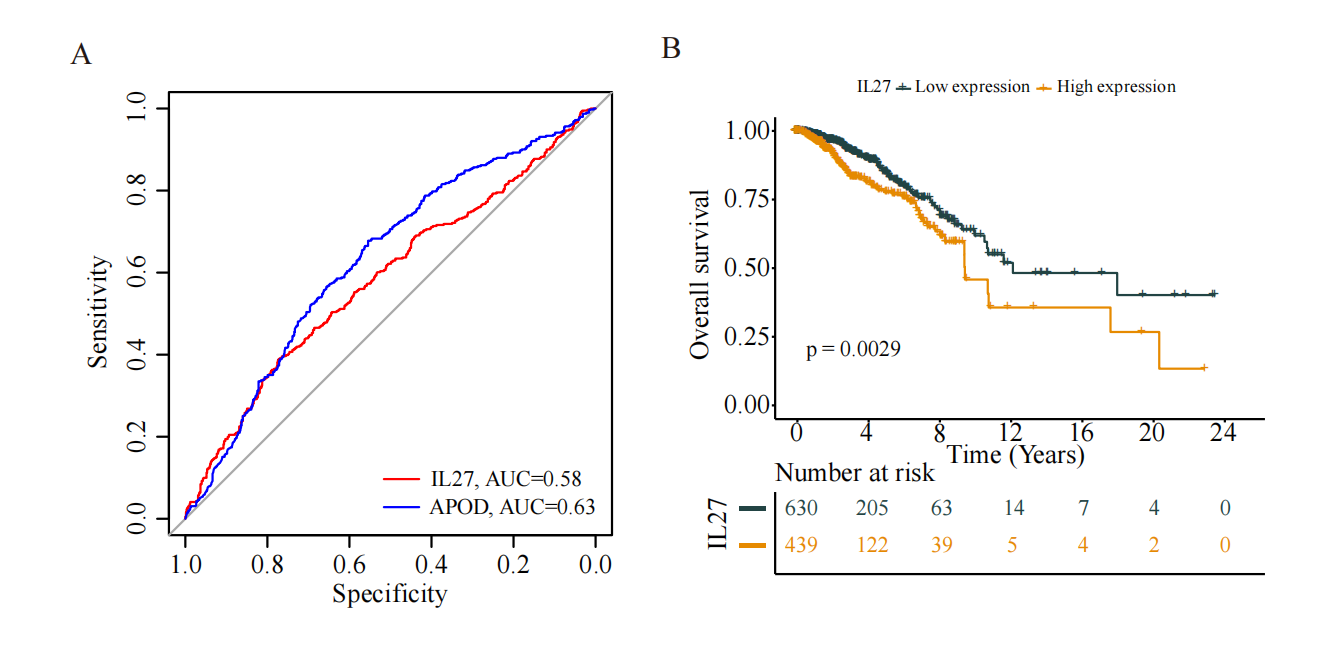

Supplement: Supplementary file 4 [file Image_4.tif]

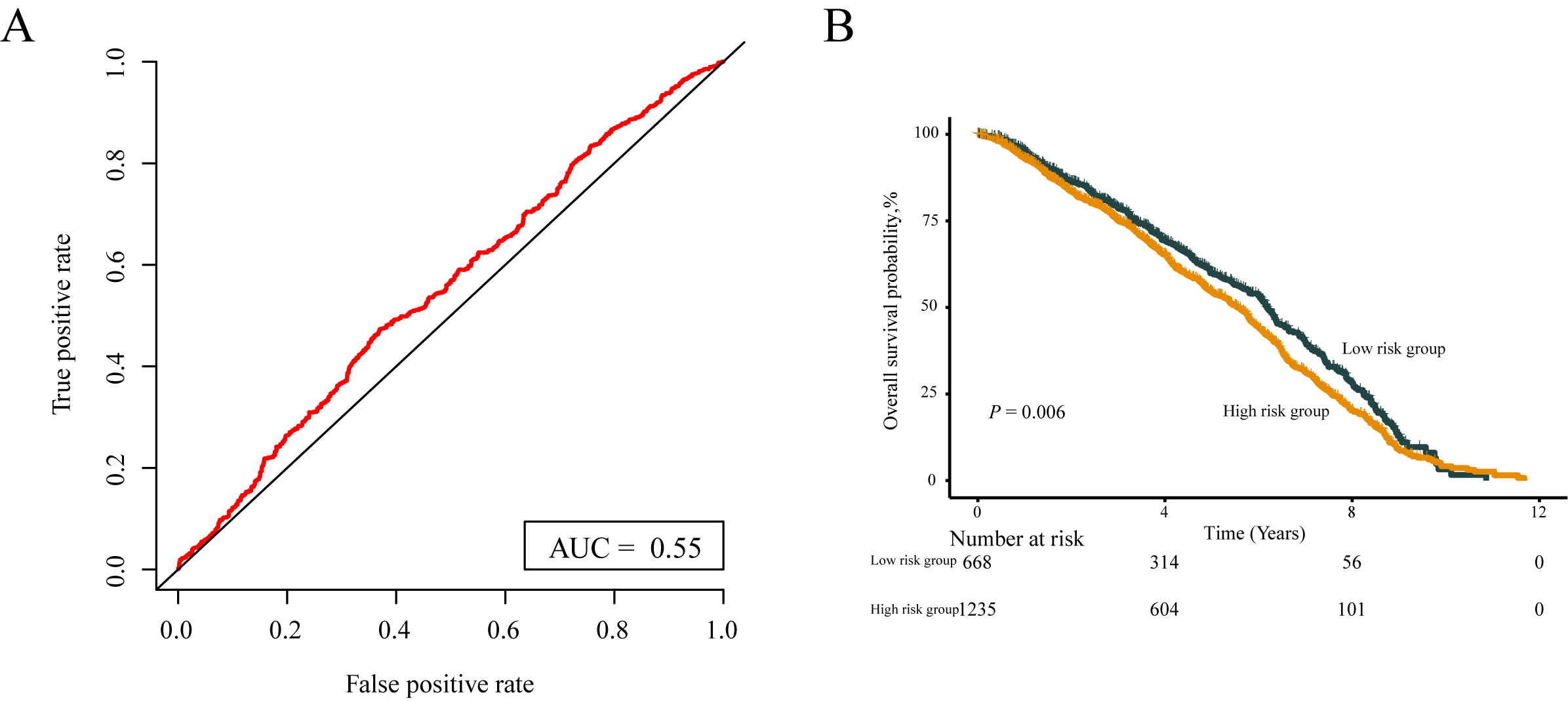

Supplement: Supplementary file 5 [file Image_5.tif]

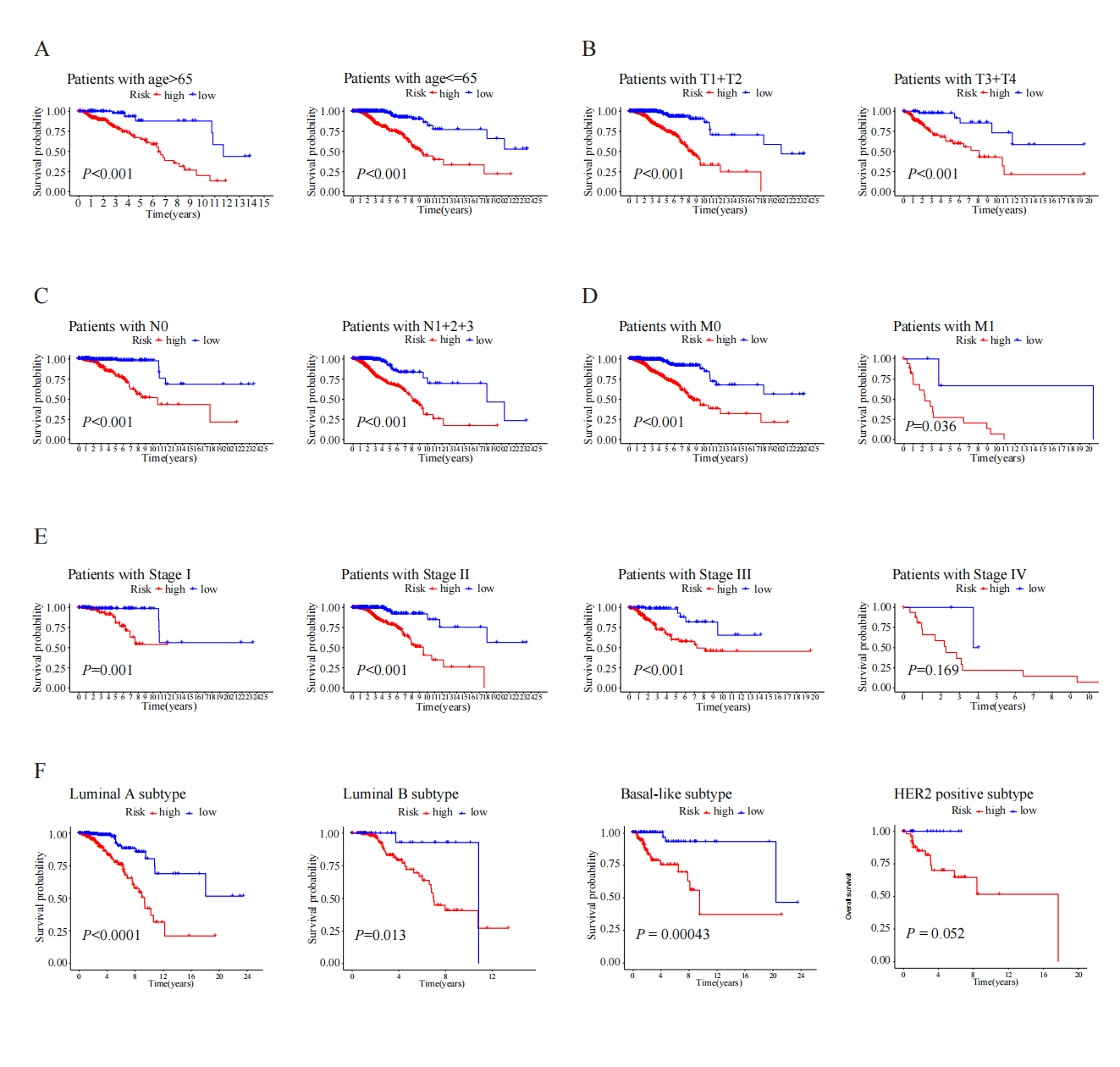

Supplement: Supplementary file 6 [file Image_6.tif]

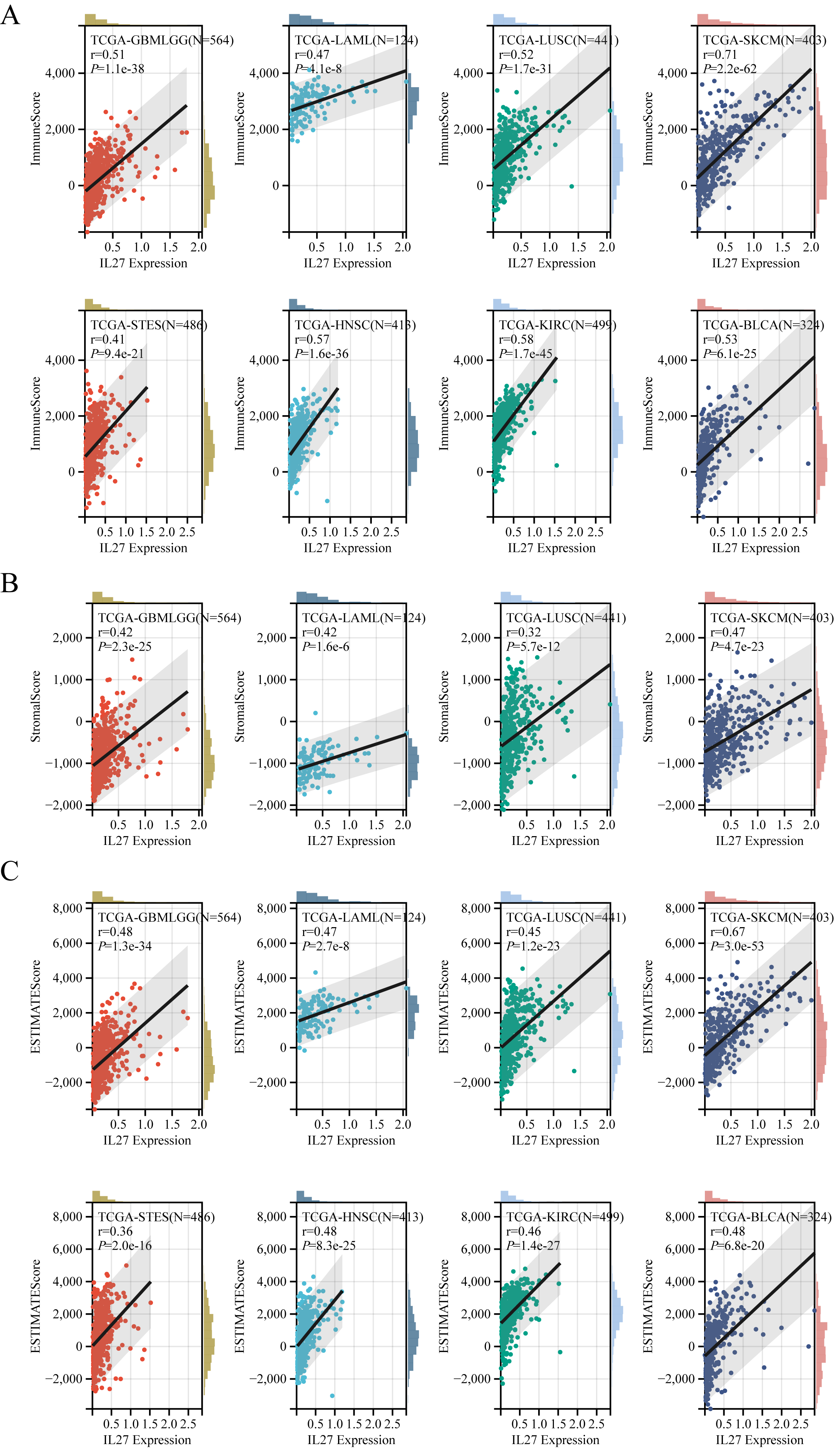

Supplement: Supplementary file 7 [file Image_7.tif]
