# Supplementary material for: Identification of a tumor immune-inflammation signature predicting prognosis and immune status in breast cancer
Source: Front Oncol. 2023 Jan 12;12:960579. doi: 10.3389/fonc.2022.960579 (PMC9881411; doi:10.3389/fonc.2022.960579)
Supplement: Supplementary file 8 [file Table_1.docx]

| **Datasets** |  | TCGA-BRCA  (N=1069) | METABRIC  (N=1903) |
| --- | --- | --- | --- |
| **Age**  (mean±SD, years) |  | 58.09±12.93 | 61.09±12.98 |
| **Status** (N) | Alive | 921(86.16%) | 800(42.04%) |
|  | Dead | 148(13.84%) | 1103(57.96%) |
| **Survival time**  (mean±SD, months) |  | 41.87±39.64 | 125.19±76.30 |
| **Clinical stage** (N) | Stage I  Stage II  Stage III  Stage IV  unknown | 181 (16.93%)  606 (56.69%)  240 (22.45%)  20 (1.87%)  22 (2.06%) | -  -  -  -  - |

**Table 1. The clinicopathologic characteristics of the TCGA-BRCA and METABRIC cohorts**
